# Conserved PCR Primer Set Designing for Closely-Related Species to Complete Mitochondrial Genome Sequencing Using a Sliding Window-Based PSO Algorithm

**DOI:** 10.1371/journal.pone.0017729

**Published:** 2011-03-18

**Authors:** Cheng-Hong Yang, Hsueh-Wei Chang, Chang-Hsuan Ho, Yii-Cheng Chou, Li-Yeh Chuang

**Affiliations:** 1 Department of Electronic Engineering, National Kaohsiung University of Applied Sciences, Kaohsiung, Taiwan; 2 Department of Network Systems, Toko University, Chiayi, Taiwan; 3 Department of Biomedical Science and Environmental Biology, Kaohsiung Medical University, Kaohsiung, Taiwan; 4 Graduate Institute of Natural Products, Kaohsiung Medical University, Kaohsiung, Taiwan; 5 Center of Excellence for Environmental Medicine, Kaohsiung Medical University, Kaohsiung, Taiwan; 6 Cancer Center, Kaohsiung Medical University Hospital, Kaohsiung Medical University, Kaohsiung, Taiwan; 7 Department of Medical Laboratory Science and Biotechnology, Chung Hwa University of Medical Technology, Tainan, Taiwan; 8 Department of Chemical Engineering & Institute of Biotechnology and Chemical Engineering, I-Shou University, Kaohsiung, Taiwan; Innsbruck Medical University, Austria

## Abstract

**Background:**

Complete mitochondrial (mt) genome sequencing is becoming increasingly common for phylogenetic reconstruction and as a model for genome evolution. For long template sequencing, i.e., like the entire mtDNA, it is essential to design primers for Polymerase Chain Reaction (PCR) amplicons which are partly overlapping each other. The presented chromosome walking strategy provides the overlapping design to solve the problem for unreliable sequencing data at the 5′ end and provides the effective sequencing. However, current algorithms and tools are mostly focused on the primer design for a local region in the genomic sequence. Accordingly, it is still challenging to provide the primer sets for the entire mtDNA.

**Methodology/Principal Findings:**

The purpose of this study is to develop an integrated primer design algorithm for entire mt genome in general, and for the common primer sets for closely-related species in particular. We introduce ClustalW to generate the multiple sequence alignment needed to find the conserved sequences in closely-related species. These conserved sequences are suitable for designing the common primers for the entire mtDNA. Using a heuristic algorithm particle swarm optimization (PSO), all the designed primers were computationally validated to fit the common primer design constraints, such as the melting temperature, primer length and GC content, PCR product length, secondary structure, specificity, and terminal limitation. The overlap requirement for PCR amplicons in the entire mtDNA is satisfied by defining the overlapping region with the sliding window technology. Finally, primer sets were designed within the overlapping region. The primer sets for the entire mtDNA sequences were successfully demonstrated in the example of two closely-related fish species. The pseudo code for the primer design algorithm is provided.

**Conclusions/Significance:**

In conclusion, it can be said that our proposed sliding window-based PSO algorithm provides the necessary primer sets for the entire mt genome amplification and sequencing.

## Introduction

Mitochondrial DNA (mtDNA) is very popular for studying evolutionary relationships [1] because of it's maternal inheritance and very high mutation rate, and lack of recombination [2], [3], [4], [5]. Although establishing the phylogenetic tree is an important tool in studying the evolutionary relationships between organisms, most organisms depend solely on a small part of the entire mtDNA, such as *cytochrome b (cyt b)* [6], [7], *cytochrome oxidase subunit I (COI)* [8], [9], and others. These studies tend to underestimate the contribution of the variation of the entire mitochondrial genome in evolutionary processes.

For example, different parts in mtDNA sequences may show different mutation rates [10]. High homology often occurs in protein-coding genes and high variability appears in non-coding sequence segments [11], [12]. Moreover, the mitochondrial protein-coding genes and the D-loop region evolve faster than *12S* and *16S rRNA* genes [13]. Hence, it is essential to address the phylogenetic relationship among species inferred from the different parts of mtDNA if the whole mt genomic sequences are available.

Although some mt genomic sequencing studies have been published [14], [15], [16], [17], [18], [19], [20], mitochondrial genome sequencing for most species is still incomplete as shown in the GenBank due to the technical problems related to the primer design. This is especially true for closely-related species. Previously, development of the conserved primers for rapid sequencing of the complete mitochondrial genome for several species, i.e. three species of bears, has been proposed [21]. However, the conserved primers were commonly designed by manual inspection of the prealigned mtDNA sequences, especially for the whole mitochondrial genome. To perform mitochondrial genome sequencing for many species without computation is still a challenge.

To solve these problems, we developed a heuristic approach, particle swarm optimization (PSO), coupled with the sliding window mechanism to design the most suitable primer sets for amplification of to several similar mtDNA sequences after multiple sequence alignments from several closely-related species. PSO and the sliding window technique constitute an optimization technique and a randomized search respectively, and derive their working principles from the social behavior of organisms. Several important criteria in primer design, including the melting temperature, the PCR product size, the secondary structure, and the uniqueness of each designed primer [22] were considered in this study. Because the unreliable sequencing data at the start end of the first 30 to 40 nucleotides (nts), our proposed algorithm was designed to avoid these problems. Our strategy is to provide the primer sets that generate the PCR amplicons for each neighboring region with partial overlap. Accordingly, the unreliable sequencing data at the start end in one PCR amplicon can be compensated for by the 3′ end sequencing data from its upstream PCR amplicon. Finally, we selected two sets of entire mt genomic sequences from two closely-related fish species to successfully demonstrate that our proposed algorithm was able to effectively identify the common primer sets for amplifying the entire mt genomic sequence.

## Materials and Methods

### Alignment of multiple sequences

**Figure 1:**
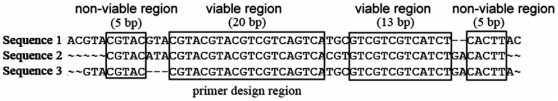
Identification of suitable primer design regions from a ClustalW generated sequence alignment (The parameter setting for the minimum primer length was 18).

Sequence alignment is the first step when designing a set of common PCR primer pairs of homologous sequences, which may be derived from closely-related species. A well-known multiple sequence alignment tool, ClustalW [23], was employed in this study. In the example of sequence alignment from three homologous sequences (Figure 1), the length of the match regions with larger than the constraint of the shortest primer length are regarded as the viable regions for primer design. After multiple sequences are aligned, numerous viable regions may be found and conserved. After the sliding window process is performed, the forward and reverse primers within the overlapping and conserved regions can easily to be designed to amplify the region between them.

### Primer design using particle swarm optimization

**Figure 2:**
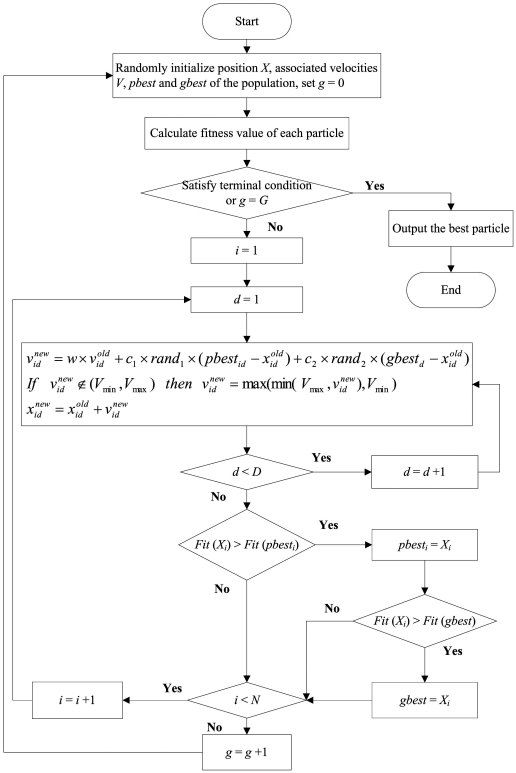
Flow chart of primer design using PSO to fit the primer design constraints.

Primer design is of crucial importance for PCR experiments. The quality of primers always influences whether a PCR experiment is successful or not. To obtain high quality primers, many primer design constraints must be satisfied. A heuristic algorithm particle swarm optimization (PSO) is employed. Particle swarm optimization is a population-based stochastic optimization technique, which was developed by Kennedy and Eberhart in 1995 [24]. PSO simulates the social behavior of organisms, such as birds in a flock and fish in a school. This behavior can be described as an automatically and iteratively updated system. In PSO, each single candidate solution can be considered “an individual bird of the flock”, that is, a particle in the search space. Each particle makes use of its own memory and knowledge gained by the swarm as a whole to find the best (i.e., optimum) solution. All of the particles have fitness values, which are evaluated by a fitness function so that they can be optimized. During movement, each particle adjusts its position by changing its velocity according to its own experience and according to the experience of a neighboring particle, thus making use of the best position encountered by itself and its neighbor. Particles move through the problem space by following a current of optimum particles. The process is then reiterated a fixed number of times or until a predetermined minimum error is achieved [24]. The computational flowchart of the PSO is provided (Figure 2). Details of the PSO algorithm are discussed is the following sections.

### Encoding schemes

**Figure 3:**
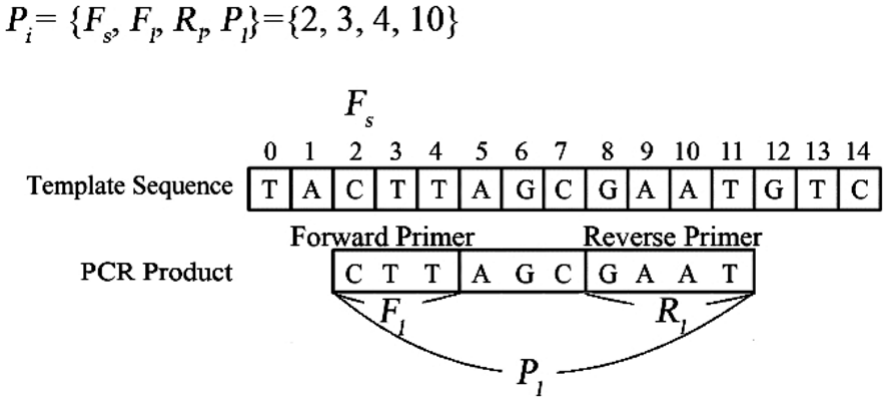
Illustration of the structure of a particle in PSO.

As stated above, we employed PSO to design suitable primer sets. In the PSO, each particle is designed in a format that enabled us to express a particular primer pair. The encoding method used was the following:
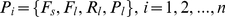
(1)where *n* represents the size of the population, *F_s_* represents the start index of the forward primer, *F_l_* represents the length of forward primer, *R_l_* represents the length of the reverse primer and *P_l_* represents the length of the PCR product. In PSO, a particle thus described represents a primer pair in a specific window. A particle represented by *P_i_* = {2, 3, 4, 10} as shown in Figure 3 as an example. Based on the template sequence, the *i*-th particle representing the PCR product is ‘CTTAGCGAAT’ in which the forward and reverse primer are ‘CTT’ and ‘ATTC’ (with complementary to reverse primer of GAAT), respectively.

### Population initialization

To initialize a population, all of the particles are randomly generated and each particle is given a velocity (*v*) within 0∼1. Initially, *F_s_* is randomly generated in the template sequence length of the window, and *F_l_* and *R_l_* are generated within the constraints of the primer length. *P_l_* is randomly generated within the constraints of the PCR length. The velocity of each dimension of each particle is randomly generated.

### Fitness function

A successful PCR experiment depends on the quality and specificity of the designed primers. The optimal primer must satisfy many criteria [25]. In PSO, a fitness function is used to evaluate the fitness of each particle in order to check whether the primer pairs satisfy the design constraints. We combined many primer design constraint functions with weights into the fitness function. The constraints used are the followings: *1) melting temperature*, *2) primer length and GC content*, *3) PCR product length*, *4) secondary structure*, *5) specificity*, and *6) terminal limitation*. These constraints are described in detail below:

#### 1) Melting temperature

To perform a successful PCR experiment, the melting temperature (*Tm*) for each primer must be confined to a suitable range. In this study, the value of the melting temperature of the primer is denoted *Tm*(*P_i_*), referring to the formula based on nearest neighbor thermodynamic theory by Freier et. al (Eq. (2)) [26], [27] and adopted from the user manual of “NetPrimer” software from PREMIER Biosoft International (http://www.premierbiosoft.com/DATAFILES/NetPrimerManual.pdf). In Eq. (2), 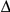
H and 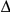
S indicate enthalpy and entropy for helix formation, respectively, which are both calculated as the nearest-neighbor model as described [28]; R is molar gas constant (1.987 cal/°C * mol); C is the nucleic acid concentration (default value 250 pM); [K^+^] is salt concentration which is equal to total [Na^+^] equivalent calculated using the concentration values of the monovalent ion and free [Mg^2+^] ion (default values 50 and 1.5 mM, respectively) (Eq. (3)). Function *Melt_tm*(*P_i_*) is used to check whether the melting temperature of a primer pair is between 54 and 65°C, where *P_iF_* and *P_iR_* denote the forward primer and reverse primer of the *i*-th particle, and the *abs()* denotes the absolute value. 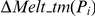 is used to check whether the difference of the melting temperature exceeds 3°C.
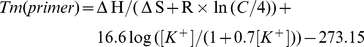
(2)

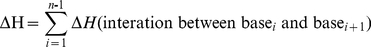
(3)which *n* is sequence selected length.
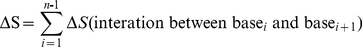
(4)which *n* is sequence selected length.
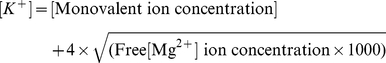
(5)

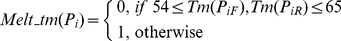
(6)

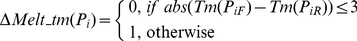
(7)


#### 2) Primer length and GC content

In general, the primer length should be within 18 and 28 nts, and the differential length of a primer pair is restricted to less than 3 nts [29]. |*P_iF_*| and |*P_iR_*| represent the number of nucleotides of the forward and reverse primers, respectively. In Eq. (8) and (9), the 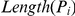 is used to check whether the length of a primer pair is within 18 to 28 nts, and 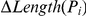 is used to check whether the length difference between the forward and reverse primers exceeds 3 nts or not.
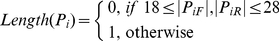
(8)

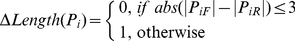
(9)The GC content check function 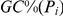 is denoted as Eq. (7)
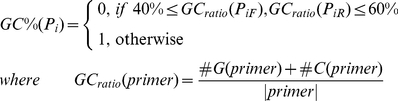
(7)


#### 3) PCR product length

MtDNA is a double-stranded circular molecule. PCR amplicons using several primer pairs are surrounded by the entire mtDNA sequences without gap. It is also essential to maintain the coverage for assembly of the individual sequences from different amplicons into the complete mt genome. The length of the PCR product has to be considered. In Eq. (10), 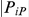 is the PCR product length. Using forward and reverse primers to perform the two-directional sequencing are helpful for double checking the sequence for 800–1100 nts in reliability. When the PCR product length setting of 800–1100 nts is not available, it is adjustable to reduce or extend the PCR length to a suitable range until it is reached.
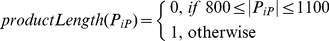
(10)


#### 4) Secondary structure

In primer design, primers that bind to any sites on the sequence indiscriminately have to be avoided. Furthermore, primers that are self-complementary (self-dimer) or complement each other (cross-dimer) must also be avoided where the dimer was defined to possess over 5 base parings and the hairpin length had to longer than 4 bps. These constraints are defined in Eq. (11) and Eq. (12):
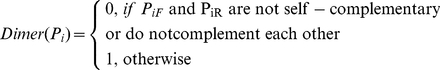
(11)

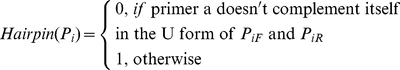
(12)


#### 5) Specificity

The specificity constraint is used to judge whether the sequence repeatedly occurs in primer or not in order to ensure the specificity of the primer. The PCR experiment might fail if the primer is not site-specific or appears more than once in the sequence.
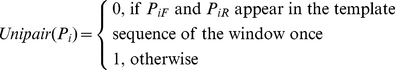
(13)


#### 6) Terminal limitation

*GC_clamp*(*P_i_*) is used to judge whether the nucleotides located in the 3′ end of primer are G or C:
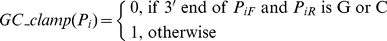
(14)


#### 7) End match

*Endmatch*(*P_i_*) is used to judge whether the three nucleotides located in the 3′ end of primer are base parings:
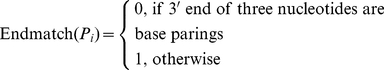
(15)


#### 8) Terminus GC number limitation

*GC_Terminus Limitation*(*P_i_*) is used to judge whether the five nucleotides located in the 3′ and 5′ termini of primer occur not over 3G or 3C:
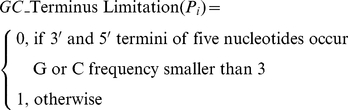
(16)


Based on the several primer design constraints described above, the fitness of each particle is evaluated by the fitness function. A low fitness value means that the particle fits more constraints. The default fitness function can be written as:
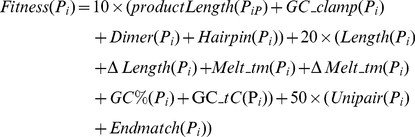
(17)


All the constraints of multiplex PCR primer design are combined into an objective function with weights. Every constraint weight setting is based on our previous researches [30], [31].

### Particle update

One of the characteristics of PSO is that each particle has a memory of its own best experience. At every iteration the particle's trajectory is updated by two “best” values, called *pbest* and *gbest*. Each particle keeps track of its coordinates in the problem space, which are associated with the best solution (fitness) the particle has achieved so far. This fitness value is stored, and represents the position called *pbest*. When a particle takes the whole population as its topological neighborhood, the best value is a global optimum value called *gbest*.

In this study, the adaptive functional values were based on the particle features representing the feature dimension; this data was evaluated by several constraints to obtain a fitness value. Once the adaptive values *pbest* and *gbest* are obtained, the features of the *pbest* and *gbest* particles can be tracked with regard to their position and velocity. Each particle is updated according to the following equations.
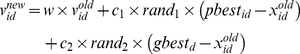
(18)

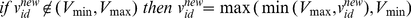
(19)

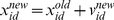
(20)


In these equations, 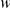 is the inertia weight, 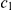 and 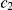 are acceleration (learning) factors, and *rand*_1_ and *rand*_2_ are random numbers between 0 and 1. Velocities 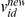 and 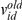 are those of the updated particle and the particle before being updated, respectively, 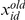 is the original particle position (solution), and 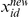 is the updated particle position (solution).

In Eq. (19), particle velocities of each dimension are tried to a maximum velocity 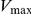. If the sum of the accelerations causes the velocity of that dimension to exceed 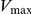, the velocity of that dimension is limited to 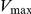. 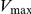 and 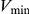 are user-specified parameters.

### Primer design based on the sliding window technique

**Figure 4:**
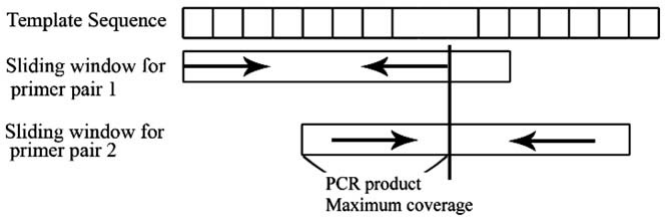
Illustration of the sliding window-based PSO algorithm for primer design. The arrow lines indicate the forward and reverse primers.

**Figure 5:**
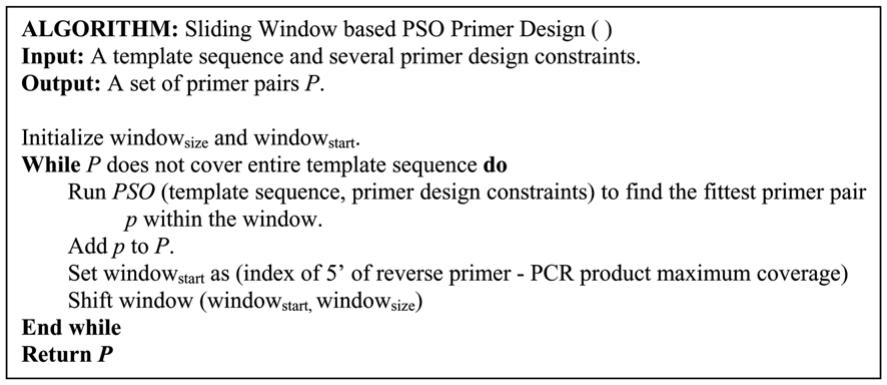
Pseudo-code of the sliding window based PSO primer design.

The sliding window method is simple and quick technique and has been widely applied in several computation fields, such as 16S ribosomal DNA amplicons in metagenomic studies [32], primer set selection in multiple PCR experiments [33], real-time primer design for DNA chips [34], and accurate location of eukaryotic protein coding regions [35]. The basic concept defines a window which is sliding across the template sequence as a specified shift value. In multiple primer design problems, the window is slid using each previous primer pair at a time along the template sequence. Subsequently, a set of primer pairs can be designed for the coverage of the entire mt genome. A detailed diagram for the sliding window method is provided (Figure 4). First, the window starts from the first nucleotide of the conserved sequences and the PSO algorithm applies it to the designed primer pair within. Then the window shifts based on the previous (first) reverse primer and the next (second) forward primer is designed from the upstream region of the first reverse primer. Each shift provides enough length for fitting PCR product coverage constraints (ranging from 90 to 200 nts of the upstream of the reverse primer). When primers can not be designed within this constrain, the overlapping length for each adjacent PCR product is extended automatically until the primers are suitable. This procedure continues until the entire template sequence is covered by all primer pair sets. The remaining primers are designed in the same way. The pseudo-code of the sliding window method based PSO primer design is provided (Figure 5). The brief JAVA 6.0-based software solution that implements our proposed algorithm was provided in the Supporting Information (Documentation S1 and Software S1).

## Results

In this research project, we applied a high-performance PSO algorithm to design fit primer pairs, and used the sliding window technique to cover the entire mt genomic sequences. The test data sets and the detailed experiment result are described below.

### Parameter settings

The termination condition of the PSO in this study is reached at a pre-specified number of iterations (in our case the number of iterations was 50). Parameters used here were a population size of 20, *rand_1_* and *rand_2_* were random numbers between (0, 1), and 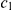 and 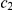 were set to 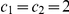. The inertia weight 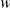 was 0.8. The velocity constraints were set to 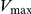 = 6 and 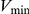 = −6.

### Test data

Entire mitochondrial genomic DNA sequences of two closely-related fish species, i.e., *Scarus forsteni* (FJ619271.1) and *Scarus rubroviolaceus* (FJ227899.1) are used as an example for designing the conserved PCR primer sets for closely-related species using a PSO algorithm. These mt genomic sequences were downloaded from NCBI GenBank.

### Dry experiment

After multiple sequences were aligned, we implemented the PSO algorithm and the sliding window technique to examine the performance on biological test data, as described above. A set of primers for amplifying entire the mt genome which was approximately 16 kb long, was designed using the proposed algorithm.

As shown in Table 1, these primers had a similar length, GC content and annealing temperature and the constraints fit the general primer design restrictions. The forward 5′-ATTTAGCCAATGACACCTAGCC-3′ and the reverse 5′-CGATTTGCACGAGTATTTTCTC-3′ of the primer pair No. 1, the length (nts), GC% and *Tm* (°C) were 22 *vs.* 22, 45.5 *vs.* 40.9 and 58.2 *vs.* 57.3, respectively. All primer sets listed in Table 1 obey the common primer constraints, i.e. hairpin, dimer and specificity. The PCR amplification was easy to complete. The adjacent PCR product overlap constraint in the proposed algorithm was set to 90∼200 nts as mentioned in the materials and methods section. The overlapping lengths are 104 ( = 1058+22−976) and 165 nts ( = 1956+25−1816) for primer sets 1 and 2 and primer sets 2 and 3, respectively. Except for the primer sets 4/5, 6/7, 7/8, 8/9, 14/15, 19/20, 21/22 and 22/1, the overlapping lengths for the other primers were all in the range of 90 to 200 nts. When suitable primer pairs in the pre-set overlap length range were unavailable, the overlapping lengths of some adjacent PCR products were raised automatically. The overlapping lengths for primer sets 4/5, 6/7, 7/8, 8/9, 14/15, 19/20, 21/22 and 22/1 for example, were 207, 256, 310, 247, 362, 301, 243 and 398 nts, respectively.

**Table 1:** A set of primers for amplifying entire circular mtDNA.

No.	Forward Primer					Reverse Primer					Product Length
	Sequence (5′ – 3′)	StartIndex*	Length	GC%	Tm	Sequence (5′ – 3′)	StartIndex*	Length	GC%	Tm	
1	ATTTAGCCAATGACACCTAGCC	193	22	45.5	58.2	CGATTTGCACGAGTATTTTCTC	1058	22	40.9	57.3	887
2	GTAACATGGTAAGTGTACCGGAAG	976	24	45.8	57.9	CGAGTTCCTTCTTTCCTTTTTAGTC	1956	25	40.0	59.9	1005
3	TGCATACGTGTACGTCGGAAC	1816	21	52.4	59.4	AGATAGAAACTGACCTGGATTGC	2619	23	43.5	57.3	826
4	TGGATCAGGACATCCTAATGGTG	2531	23	47.8	61.3	GTTTCGGCCAGGGTGGAAAT	3426	20	55.0	63.4	915
5	TTCAAAATATGCCCTCATCGG	3239	21	42.9	60.2	AAGTATTTGGCCGTTGCTTCTAC	4278	23	43.5	60.1	1062
6	CTAGGAACCACAATCACATTCG	4164	22	45.5	57.4	GCAAGTTTTAGTTCAGGGTCTG	5229	22	45.5	56.4	1087
7	TACCTCCGCCTCTCATACGC	4995	20	60.0	60.2	CATATTGTTCATTCGAGGGAAGG	5842	23	43.5	60.6	870
8	CATCCTACCTGTGGCAATCACAC	5555	23	52.2	61.8	CATCATGGCTCAGACCATGCC	6378	21	57.1	63.3	844
9	CCTCTCACTTCCTGTCCTTG	6152	20	55.0	54.2	AGCTGTGTAATAGCTTGCTTTAC	7200	23	39.1	54.0	1071
10	AGAAAGGAAGGAATCGAACCC	7118	21	47.6	59.2	TAGTGCCATCGTCAGGATCAG	8182	21	52.4	58.3	1085
11	GAATGGTGGCTCCCAATCAC	8006	20	55.0	59.7	TTGATGTGCCATTAGACGTTTTC	8866	23	39.1	59.7	883
12	CTAATCGCAACAGCCGTTTTC	8713	21	47.6	60.3	AGACCCGGTGATTGGAAGTCAC	9689	22	54.6	62.6	998
13	CCACTTTGGCTTTGAAGCAG	9569	20	50.0	58.5	TGGGTTGAGATGTGTTCATCC	10590	21	47.6	57.7	1042
14	ACCGCCTAAAAAACCTAAACC	10418	21	42.9	57.7	TTGGGAGAGGATTCCTGCTAC	11352	21	52.4	58.4	955
15	CTTTCACCTCCACATCATATGC	11011	22	45.5	57.2	GGATTTGCACCAAGAGTTTTTG	12029	22	40.9	59.3	1040
16	AAGACGCTAGGTTGTGATTCTAG	11857	23	43.5	55.6	CATGTTGTGAGGGCTGTCTG	12890	20	55.0	56.6	1053
17	CTACTTCACTCAAGCACTATGGTTG	12815	25	44.0	58.3	GTTAAATTGTTTGGCTGTTAGTGATG	13593	26	34.6	59.9	804
18	TCCCATTAAAAATCCCAGTC	13497	20	40.0	54.3	TTCGGAGACTTGCCATTAATAG	14487	22	40.9	57.0	1012
19	ACGGATTAGAAGCAACCCCAAT	14349	22	45.5	61.9	ATAAGGAGGGCTGCAAATCCTAG	15181	23	47.8	61.3	855
20	AAATATCCTTCTGAGGTGCAACC	14903	23	43.5	59.5	GAGCTAGAGGTGGAGGTTAAAATC	15717	24	45.8	58.2	838
21	GCTTAATATAAAGCACCGGTCTTG	15644	24	41.7	60.0	CTTCAGTGTTATGCTTTTGTTAAGC	9	25	36.0	58.2	1092
22	ACTTGAGTTTCCCCCCTACCC	16470	21	57.1	61.0	TCCTTTGGGTTTTAAGCTTACGCT	567	24	41.67	63.27	823

*The position of the first nucleotide in primer. The nucleotide “1” is the first nucleotide in the GenBank accession nos as described in Section materials and methods.

**Figure 6:**
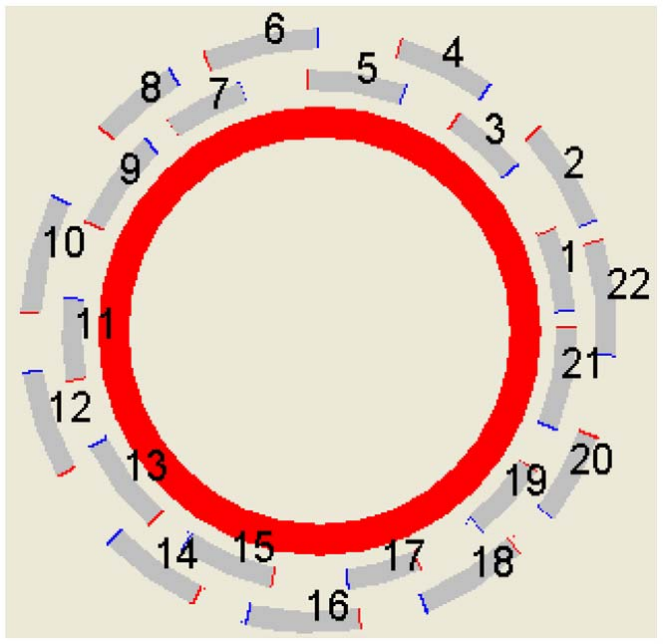
A set of conserved primer pairs for sequencing an entire mitochondrial genome.

In order to amplify the entire mt genome, it is essential to design a group of primer pairs that are partly overlapping each other. Based on the sliding window technique, the proposed algorithm can easily design primers that to allow PCR amplification for 22 adjacent PCR products that overlap each other. As shown in Figure 6, the proposed algorithm yields the entire sets of forward and reverse primers. The expected length for these PCR products ranged from 800 to 1100 nts, and these primer sets are arranged neatly and overlap with the adjacent PCR products. All designed forward and reverse primers of each PCR product are designed within the conserved regions of the multiple sequence alignment.

### Wet experiment

**Figure 7:**
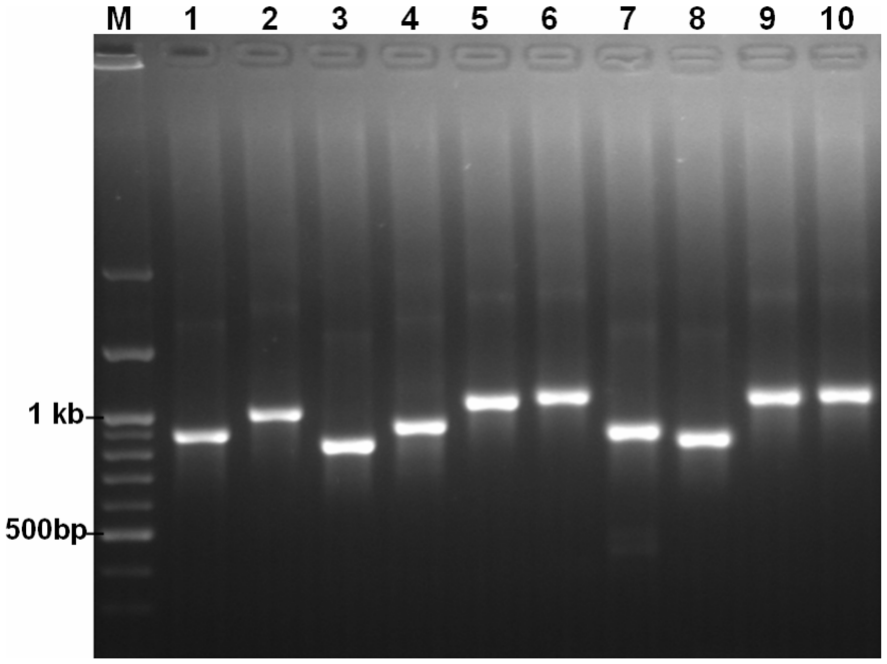
Demonstration of the PCR performance for ten sets of designed conserved primers. Lanes 1 to 10 are the PCR amplification using primer sets 1 to 10 listed in the Table 1.

The first ten primer sets for mitochondrial genome of *Scarus forsteni* provided in Table 1 were examined by PCR amplification. DNA samples (200 ng) were added to the PCR reaction mixture (10 µl) containing 1 µl of 10× PCR buffer, 0.3 µl of 50 mM MgCl_2_, 0.2 µl of 10 mM dNTP each, 0.6 µl of DMSO, 0.14 µl of 5 U Platinum Taq enzyme (Invitogen corp.), 0.12 µl of 350 µg/ml primer mix (1∶1), and 7.64 µl of DNA in water. The touch-down PCR program [36] was applied to all the test primer sets (listed in Table 1, sets 1 to 10) and it was slightly modified as follows: 94°C (3 min); 3 cycles of 94°C (15 s), 63°C (15 s), 70°C (30 s); 3 cycles of 94°C (15 s), 60°C (15 s), 70°C (30 s); 3 cycles of 94°C (15 s), 57°C (15 s), 70°C (30 s); 49 cycles of 94°C for (15 s), 54°C (15 s), 70°C (30 s). To avoid the very high risk of sample mixup and the resulting artificial recombination when using e.g. many different primer pairs (see Table 1), however, parallel amplification of all targets per specimen would be highly recommended. As shown in Figure 7, the designed primer sets were successfully amplified by PCR.

## Discussion

The public resources available for mitochondrial bioinformatics have been reviewed [37]. Among them, only V-MitoSNP [36] provides primer design for mitochondrial genome sequences. However, V-MitoSNP is still limited to mtSNPs. Currently, several primer design algorithms are used, e.g. genetic algorithm (GA) [29], [38], heuristic algorithm [25], memetic algorithm (MA) [30], PSO algorithm [31], and consecutive multiple discovery (CMD) algorithm [39]. However, these algorithms were developed for primer design without considering the situations in multi-sequence alignment, and for the circular sequences like the mt genome. In contrast, some sequence alignment tools, such as CLUSTALW [40], TBA [41], MAVID [42], MLAGAN [43], Pecan [44], Seq-SNPing [45], AQUA [46], and Cgaln [47], were developed without a primer design function. Accordingly, the development of integrated algorithms for designing feasible primer sets for entire mt genome sequences coupled with the sequence alignment are still challenging.

Recently, GeneFisher2 [48] was developed to design a number of primer pairs for consensus sequence after multi-sequence alignment. However, like most primer design tools, GeneFisher2 only focuses on designing a primer pair or a number of primer pairs on a limited region and does not design a set of primer pairs for amplifying an entire sequence at once. Primer design for amplification and sequencing of an entire mt genome is currently under development.

PSO has been reported to progress faster than genetic algorithm (GA) with crossover, mutation, or both [49]. In our previous work for primer design [31], we found that the average accuracy of PSO was better than memetic algorithm (MA) [30] and genetic algorithm (GA) based on the Wallace formula [50] (100% *vs.* 98.33% and 74.93%) and the Bolton and McCarthy formula [51] (94.93% *vs.* 88.93% and 32.40%) for five hundred runs with PCR product length between 150∼300 nts, 500∼800 nts and 800∼1000 nts. Therefore, we applied the PSO rather than the MA and GA to design the whole set of primers for the whole mt genomic sequencing.

In this study, we propose a novel strategy that introduces the sliding window technique coupled with a PSO algorithm. This algorithm provides a sequence alignment function for the entire circular genome and designs a set of primers that generates overlapping PCR amplification for the entire circular genome, e.g. the mt genome. Several primer design constraints for a successful PCR were considered in the proposed algorithm. For example, the melting temperature, the primer pair length, the GC content, the PCR product length, the secondary structure, and the specificity were all considered. The primer design results demonstrated that a set of primers that obeys these design constrain with suitable length and could be found for the PCR amplicons. Other circular genome sequences such as chloroplasts and bacterial chromosomes could be used with this strategy as well.

Some entire mt genome sequences are available for some species in GenBank, and hence they requirement for sequencing is reduced. However, the known mtDNA sequences could be applied to the entire mt genomic sequences of closely-related species based on our experience. For the purpose of sequencing species with an unknown mt genomic sequence, we suggest to collect the entire mt genomic sequences of the same family or order and perform our proposed algorithm. Subsequently, the designed primer sets derived from the conserved regions stand a high possibility of being used to perform successful PCR reactions for the entire mt genomic sequencing to the species with unknown mtDNA sequence.

Utilizing the heuristic algorithm and sliding window technique, we used several primer constraints to appraise the fitness values and, based on their respective significance, each constraint was given a corresponding weight. Through the design of a fitness function, our algorithm was able to design a complete set of primers. The strategy of this algorithm was designed to carefully select a set of common PCR primer pairs from the conserved regions by multiple nucleotide sequence alignment. The common PCR primer pairs designed by our algorithm could be applied to amplify conserved sequences of genes from the same or different organisms. It can be used to amplify conserved sequences of genes from other gene families. For example, the whole primer sets for amplifying the entire circular mtDNA for Chimpanzees (*Pan troglodytes*; accession no. NC 001643) [52] and Bonobo (*Pan paniscus*; accession no. GU189657) [53] are successfully mined using our proposed methodology (data not shown). In our study, two fish mitochondrial genome data sets were used to test the proposed method and ten of the whole primer sets were successfully amplified to prove the performance of this algorithm. Taken together, our proposed primer design algorithm is an effective method to design primer pair sets for PCR amplification and sequencing for the multiple alignments of circular consensus sequences. The entire mt genome sequencing was easy to complete and could help biologists in identifying the phylogenetic relationship for closely-related species.

## Supporting Information

Documentation S1 (DOC)

Software S1 (RAR)
