# Supplementary material for: Conserved PCR Primer Set Designing for Closely-Related Species to Complete Mitochondrial Genome Sequencing Using a Sliding Window-Based PSO Algorithm
Source: PLoS One. 2011 Mar 18;6(3):e17729. doi: 10.1371/journal.pone.0017729 (PMC3060828; doi:10.1371/journal.pone.0017729)
Supplement: Documentation S1 — (DOC) [file pone.0017729.s001.doc]

**Document S1.** A brief Java-based software (see the software S1 in the supporting information section) which is implemented by our proposed algorithm has to operate under the Java Runtime Environment (JRE). After de-zipping, user can start the function by clicking the file “mitoPrimer_V1”. User manual and two sample whole mt genome sequences (*Scarus_rubroviolaceus* and *Scarus forsteni*) mentioned in this study are available in the software.
